# Child Abuse Knowledge, Empathy, and Attitudes Toward Reporting Child Abuse Among Nurses Working in Children’s Hospitals in South Korea: The Mediating Role of Child Abuse Reporting Self-Efficacy

**DOI:** 10.3390/healthcare14213665

**Published:** 2026-10-28

**Authors:** SoRi Kim, EunJung Kim

**Affiliations:** Department of Nursing, Honam University, Jeonnam-Gwangju Special Metropolitan City 62399, Republic of Korea; ksori@kentech.ac.kr

**Keywords:** child abuse, mandatory reporting, self-efficacy, empathy, nurses, mediation analysis

## Abstract

**Highlights:**

**What are the main findings?**

- In separate mediation models, child abuse reporting self-efficacy showed significant indirect associations linking child abuse knowledge and empathy to attitudes toward reporting child abuse.
- The direct association of empathy with reporting attitudes remained significant after accounting for self-efficacy, whereas that of child abuse knowledge was not statistically significant.

**What are the implications of the main findings?**

- The findings suggest that nursing education should address reporting confidence alongside child abuse knowledge and empathy.
- Longitudinal and intervention studies are needed to determine whether strengthening reporting self-efficacy and empathy improves reporting attitudes and actual reporting behavior.

**Abstract:**

**Background/Objectives**: Nurses play an important role in recognizing and reporting suspected child abuse. However, the role of child abuse reporting self-efficacy in the relationships of child abuse knowledge and empathy with reporting attitudes remains insufficiently understood among nurses working in children’s hospitals. This study examined these relationships and tested the indirect associations of knowledge and empathy with reporting attitudes through self-efficacy. **Methods**: This cross-sectional study included 173 nurses recruited through convenience sampling from seven children’s hospitals in a metropolitan city in South Korea. Data were collected using structured self-report questionnaires. Descriptive statistics, independent *t*-tests, one-way analysis of variance, and Pearson correlations were calculated. Separate mediation models were tested using Hayes’ PROCESS macro Model 4, and an integrated model including both predictors was additionally estimated, with 10,000 percentile bootstrap resamples. Sensitivity analyses adjusted for clinical experience and prior child abuse-related education. **Results:** Child abuse reporting self-efficacy was positively associated with knowledge, empathy, and reporting attitudes. In both the separate and integrated models, significant indirect associations through self-efficacy were observed for knowledge (integrated model: β = 0.08, 95% CI [0.01, 0.15]) and empathy (β = 0.11, 95% CI [0.03, 0.22]), and these findings were consistent after covariate adjustment. The direct association between knowledge and reporting attitudes did not reach statistical significance after accounting for self-efficacy, whereas the direct association between empathy and reporting attitudes remained significant. **Conclusions**: The findings suggest that child abuse reporting self-efficacy may help explain the associations of knowledge and empathy with reporting attitudes. Educational strategies addressing reporting confidence and empathy warrant further investigation. Longitudinal and intervention studies are needed to establish temporal relationships and evaluate effects on actual reporting behavior.

## 1. Introduction

Child abuse is a serious social problem that threatens not only children’s health and welfare but also their lives. Beyond the immediate harm inflicted, children who experience abuse are more likely to face adverse consequences throughout their development, including depression, obesity, and alcohol and substance misuse, as well as broader behavioral, physical, and mental health disorders. They are also more likely to perpetrate or become victims of violence later in life [1]. In the United States, approximately 3.73 million reports of child abuse and neglect were received in 2022, highlighting the substantial burden of child maltreatment [2]. In South Korea, the number of reports of suspected child abuse increased from 42,251 in 2020 to 46,103 in 2022, 48,522 in 2023, to 50,242 in 2024 [3].

Children experiencing abuse may have difficulty disclosing their experiences or seeking help, making early recognition and appropriate reporting by adults who interact with them particularly important [4]. To facilitate the early detection and reporting of child abuse, South Korea enacted the Act on Special Cases Concerning the Punishment of Child Abuse Crimes in 2014, strengthening the legal framework for responding to child abuse [5]. Healthcare professionals, who may encounter suspected child abuse in clinical practice, play an important role as legally mandated reporters. Nevertheless, healthcare professionals in Korea account for fewer than 1% of reports of suspected child abuse, a proportion that has remained persistently low for over a decade [3]. International data also underscore the important role of healthcare professionals in child abuse reporting. In the United States, approximately 26% of infant child welfare investigations between 2010 and 2019 originated from reports by medical professionals [6], while medical or health personnel accounted for 14% of known notification sources among investigated child protection cases in Australia [7]. Although these figures refer to different populations and reporting systems and are not directly comparable, they illustrate the contribution of healthcare professionals to child protection. Reasons cited for not reporting suspected abuse despite awareness include uncertainty about evidence, discomfort with legal procedures, fear of negative consequences, misattribution of abuse as parental discipline, and fear of blame/retaliation following a report [8].

Nurses working in children’s hospitals are well positioned to observe children’s clinical conditions, communicate with caregivers, and monitor parent–child interactions. These opportunities may support the recognition of suspected abuse and an appropriate response [9]. However, research involving nurses working in children’s hospitals—who are highly likely to encounter abused children in practice—remains limited, with many prior studies focusing instead on nursing students or emergency department nurses [10]. Examining reporting attitudes in this clinical population may therefore help identify factors relevant to nurses’ responses to suspected child abuse.

Previous studies have examined associations of child abuse knowledge, awareness of child abuse, and awareness of children’s rights with reporting attitudes and intentions [11,12,13]. However, reporting attitudes, reporting intentions, and actual reporting behavior are distinct outcomes. Understanding factors associated with favorable reporting attitudes is therefore an important component of research on nurses’ responses to suspected abuse [13,14,15]. Self-efficacy for reporting child abuse—defined as an individual’s belief in their capability to successfully recognize suspected abuse and take appropriate reporting action [16]—has been proposed as a key psychological factor associated with child abuse reporting behavior and actively studied abroad; however, research addressing self-efficacy for reporting child abuse remains limited in Korea. This construct is grounded in Bandura’s self-efficacy theory, which posits that an individual’s belief in their capability to successfully perform a specific behavior influences whether that behavior is initiated and sustained [17]. Thus, adequate knowledge of child abuse may not necessarily be accompanied by confidence in recognizing suspected abuse or completing reporting procedures. Previous studies have similarly identified self-efficacy as an important factor associated with child abuse reporting intention [4].

Empathy is also considered an essential competency for nurses working in children’s hospitals, who must understand children with varying developmental needs and respond appropriately to their emotional states [18]. Previous studies among nursing students have examined empathy in relation to child abuse reporting intention [13]. Research on empathy and moral sensitivity also suggests its relevance to ethical nursing practice [19].

From a broader psychological perspective, empathy has been associated with prosocial behavior and the perceived seriousness of harmful or delinquent acts. A meta-analysis demonstrated positive associations between both cognitive and affective empathy and prosocial behavior [20]. Furthermore, a cross-cultural study found that empathy significantly predicted both prosocial behavior and perceptions of the seriousness of delinquent acts [21]. These findings suggest that empathy may provide a relevant theoretical basis for understanding nurses’ sensitivity to suspected child abuse and their attitudes toward protective action. However, prosocial behavior and perceived offense seriousness are conceptually distinct from attitudes toward reporting child abuse.

Beyond individual psychological characteristics, previous research has identified associations between social support, resilience, and prosocial behavior [22]. Studies of nursing practice have also highlighted the relevance of professional competencies, clinical experience, and supportive work environments to nurses’ professional performance and the quality of care they provide [23]. In the context of child protection, these perspectives suggest that nurses’ responses to suspected abuse may be understood within a broader framework encompassing individual psychological resources, professional competence, and organizational conditions. However, these additional factors were not directly examined in the present study.

Taken together, child abuse knowledge may provide a cognitive foundation for recognizing suspected abuse, while empathy may support sensitivity to children’s experiences and motivation to protect them. Reporting self-efficacy, in turn, reflects confidence in recognizing suspected abuse and carrying out appropriate reporting procedures. Drawing on Bandura’s self-efficacy theory, reporting self-efficacy may help explain the associations of both knowledge and empathy with reporting attitudes. Nevertheless, these proposed relationships should be understood as theoretical hypotheses rather than established causal mechanisms.

Despite the potential relevance of these cognitive, affective, and psychological factors, few studies have examined their indirect associations with reporting attitudes among nurses working in children’s hospitals. Accordingly, this study aimed to examine the relationships among child abuse knowledge, empathy, child abuse reporting self-efficacy, and attitudes toward reporting child abuse among nurses working in children’s hospitals in South Korea, and to test the indirect associations of knowledge and empathy with reporting attitudes through child abuse reporting self-efficacy in separate and integrated mediation models. The findings may inform the development of educational strategies to support nurses in recognizing and responding to suspected child abuse.

## 2. Materials and Methods

### 2.1. Study Design and Participants

This cross-sectional descriptive survey study examined the relationships among child abuse knowledge, empathy, child abuse reporting self-efficacy, and attitudes toward reporting child abuse among nurses working in children’s hospitals. It also examined the indirect associations of knowledge and empathy with reporting attitudes through child abuse reporting self-efficacy in separate and integrated mediation models. This study is reported in accordance with the Strengthening the Reporting of Observational Studies in Epidemiology (STROBE) guidelines.

Participants were nurses working at seven children’s hospitals in G Metropolitan City, South Korea. Participants were recruited using convenience sampling, and data were collected using a structured questionnaire between July 2025 and August 2025. After approval from the nursing department of each hospital, the researcher explained the purpose of the study to eligible nurses, distributed the questionnaires to those who agreed to participate, and collected the completed questionnaires in sealed envelopes. To protect the anonymity of participants and institutions, the name of each participant’s hospital was not recorded on the questionnaire.

The inclusion criteria were as follows: (1) nurses currently working at one of the seven participating children’s hospitals and providing direct patient care and (2) nurses who understood the purpose of the study and voluntarily agreed to participate. Nurses working exclusively in administrative positions without direct patient care responsibilities were excluded.

A total of 180 questionnaires were collected. Of these, seven (3.9%) with partially missing responses were excluded, leaving 173 questionnaires for the final analysis. There were no missing values on any of the study variables in the final sample; therefore, no imputation was performed. Items that applied only to a subset of participants (e.g., the number of reports among those with reporting experience) were treated as not applicable rather than missing.

### 2.2. Measures

#### 2.2.1. Child Abuse Knowledge

Child abuse knowledge was measured using a tool developed as part of the Child Abuse Report Intention Scale by Feng and Levine [24] and translated and adapted into Korean by Kim [25]. The instrument comprises 49 items across two subdomains: 15 items assessing child abuse knowledge and mandatory reporting legislation, and 34 items assessing evidence of child abuse knowledge. Each item is answered as “yes” or “no,” with correct responses scoring 1 point and incorrect or “don’t know” responses scoring 0 points, yielding a total score ranging from 0 to 49; higher scores indicate greater child abuse knowledge. The Kuder–Richardson 20 (KR-20) reliability coefficient was 0.82 in Feng and Levine’s study [24], 0.83 in Kim [25], and 0.77 in the present study.

#### 2.2.2. Empathy

Empathy was measured using the Korean version of the Questionnaire of Cognitive and Affective Empathy, originally developed by Reniers et al. [26] for British university students and translated and adapted into Korean by Kim [25]. The original instrument comprised 31 items across two domains and five subfactors; based on reliability and factor analyses, Kim [25] removed five items, yielding a final 26-item Korean version measuring cognitive empathy (perspective-taking and online simulation) and affective empathy (emotion contagion, proximal responsivity, and peripheral responsivity). Items 1 and 24 were reverse-scored. Each item is rated on a 4-point Likert scale ranging from 1 (strongly disagree) to 4 (strongly agree), with higher scores indicating greater empathy. Cronbach’s α for the subfactors ranged from 0.65 to 0.85 in the original study by Reniers et al. [26], was 0.86 in Kim’s study [25], and 0.88 in the present study.

#### 2.2.3. Child Abuse Reporting Self-Efficacy

Child abuse reporting self-efficacy was measured using a tool developed by Greytak [27] and translated and adapted by Kim and Lee [28]. The instrument comprises eight items across two subdomains: four items assessing confidence in recognizing suspected cases of child abuse by type, and four items assessing confidence in reporting suspected cases of child abuse by type. Each item is rated on a 5-point Likert scale ranging from 1 (strongly disagree) to 5 (strongly agree); higher scores indicate greater child abuse reporting self-efficacy. Cronbach’s α was 0.86 in Kim and Lee’s study [28] and 0.90 in the present study.

#### 2.2.4. Attitudes Toward Reporting Child Abuse

Attitudes toward reporting child abuse were measured using a tool developed by Lee [14]. The instrument comprises 17 items across five subdomains: perceived disadvantages of reporting (four items), perceived impact of reporting on the child (four items), discomfort with reporting (four items), advocacy for the child (two items), and avoidance of reporting responsibility (three items). Items 1, 2, 3, 12, and 13 were reverse-scored. Each item is rated on a 5-point Likert scale ranging from 1 (strongly disagree) to 5 (strongly agree); higher scores indicate a more favorable attitude toward reporting child abuse. Cronbach’s α was 0.85 in Lee’s study [14] and 0.83 in the present study.

### 2.3. Data Collection

Data were collected from 14 July to 14 August 2025 from nurses working at seven children’s hospitals located in G Metropolitan City, South Korea, using a structured questionnaire. Prior to data collection, the study purpose and procedures were explained to the nursing department of each hospital by telephone and in person. After obtaining approval from the head of each nursing department, the researcher distributed the questionnaires directly to participating nurses and collected the completed questionnaires in sealed envelopes. To protect the anonymity of participants and institutions, the name of each participant’s hospital was not recorded on the questionnaire.

### 2.4. Sample Size

The required sample size was calculated using G*Power version 3.1.9.7. For multiple regression analysis, assuming a significance level of 0.05, an effect size of f^2^ = 0.15, a statistical power of 0.90, and 12 predictors, the minimum required sample size was estimated to be 157. To allow for nonresponse or incomplete questionnaires, 180 questionnaires were distributed, all of which were returned (100% response rate). After excluding eight questionnaires with incomplete responses, data from 173 participants were included in the final analysis.

### 2.5. Data Analysis

Data were analyzed using IBM SPSS Statistics for Windows, version 28.0 (IBM Corp., Armonk, NY, USA) and the PROCESS macro. Participants’ general characteristics were summarized using frequencies, percentages, means, and standard deviations. Descriptive statistics for the study variables (child abuse knowledge, empathy, child abuse reporting self-efficacy, and attitudes toward reporting child abuse) were calculated as means, standard deviations, minimum values, and maximum values.

Differences in the study variables according to participants’ general characteristics were analyzed using independent *t*-tests for variables with two categories and one-way analysis of variance (ANOVA) for variables with three or more categories, with the Scheffé test used for post hoc comparisons. The relationships among child abuse knowledge, empathy, child abuse reporting self-efficacy, and attitudes toward reporting child abuse were examined using Pearson’s correlation coefficients.

Prior to the mediation analysis, regression assumptions were examined. Multicollinearity among the independent variables was assessed using tolerance and the variance inflation factor (VIF). Residual independence, normality, homoscedasticity, and potentially influential observations were evaluated using the Durbin–Watson statistic, residual plots, standardized residuals, and Cook’s distance.

Two separate mediation models were first estimated using Hayes’ SPSS PROCESS macro Model 4 [29] to examine the indirect associations of child abuse knowledge and empathy with attitudes toward reporting child abuse through child abuse reporting self-efficacy. Child abuse knowledge was specified as the predictor in Model 1, and empathy was specified as the predictor in Model 2. The separate models were used to examine the theoretically distinct roles of knowledge as a cognitive factor and empathy as an affective factor, rather than on the basis of the nonsignificant correlation between the two variables.

To examine whether these associations remained when both predictors were considered simultaneously, an additional integrated mediation model was estimated. Child abuse knowledge and empathy were entered simultaneously as predictors in both the mediator equation, in which child abuse reporting self-efficacy was regressed on knowledge and empathy, and the outcome equation, in which attitudes toward reporting child abuse were regressed on knowledge, empathy, and self-efficacy. This specification allowed the indirect association of each predictor to be estimated while accounting for the other.

As sensitivity analyses, the separate and integrated models were re-estimated after adjustment for two potential confounders: total years of clinical experience (continuous) and prior completion of child abuse-related education (1 = yes, 0 = no). Both covariates were entered in the mediator equation and in the outcome equation of each model. The unadjusted and adjusted results, including the coefficients of the covariates, were reported. 

Indirect associations were estimated as the product of the a path (predictor → self-efficacy) and the b path (self-efficacy → attitudes), and their statistical significance of the indirect effects was evaluated using percentile bootstrapping with 10,000 resamples and 95% confidence intervals (CIs); an indirect effect was considered statistically significant if the 95% CI did not include zero. Both unstandardized (B) and standardized (β) coefficients are reported. Each indirect association is reported as an unstandardized indirect effect (B) with its bootstrap standard error (BootSE) and 95% bootstrap CI, together with the completely standardized indirect effect (β) and its 95% bootstrap CI. A nonsignificant direct association was not interpreted as evidence of full mediation. Because the data are cross-sectional, the mediation analyses tested statistical indirect associations and cannot establish temporal ordering or causal mechanisms. The terms “predictor,” “mediator,” and “outcome” therefore denote statistical roles only, and the term “indirect association” is used throughout in place of “indirect effect” as labeled in the PROCESS output. A two-tailed *p* value < 0.05 was considered statistically significant for all analyses.

### 2.6. Ethical Declarations

This study was approved by the Institutional Review Board of Honam University (Approval No. 1041223-202506-HR-17) and was conducted in accordance with the Declaration of Helsinki and relevant institutional guidelines. Permission to use each research instrument was obtained via email from the respective developers before data collection. Data collection at each hospital was performed only after formal approval was obtained in accordance with each institution’s internal regulations. Before participation, nurses received an explanation of the study purpose and procedures and were informed that participation was voluntary and that declining to participate or withdrawing would entail no penalty. Participants were also informed that anonymity would be maintained and that all collected data would be managed directly by the researcher to ensure confidentiality. Written informed consent was obtained only from nurses who agreed to participate. Completing the questionnaire took approximately 15–20 min, and participants received a small token of appreciation for their participation. Participants were informed that the collected data would be used solely for the purposes of this study, would be retained for three years after study completion in accordance with bioethics-related regulations, and would thereafter be destroyed in accordance with Article 16 of the Enforcement Decree of the Personal Information Protection Act.

## 3. Results

### 3.1. General Characteristics of Participants

In total, 173 participants were included in the analysis. The mean age of participants was 36.92 ± 8.03 years, with those aged ≥40 years accounting for the highest proportion (74, 42.8%), followed by those aged 30–39 years (62, 35.8%) and those aged ≤29 years (37, 21.4%). The mean total clinical work experience was 11.45 ± 7.46 years, with 15 years or more being the most common category (60, 34.7%). The mean work experience in a children’s hospital was 8.29 ± 6.89 years, with less than three years being the most common category (49, 28.3%). Regarding marital status, married participants accounted for the largest proportion (99, 57.2%), followed by single (69, 39.9%) and divorced (5, 2.9%) participants. Participants with children accounted for 88 (50.9%) of the sample. Regarding position, 139 participants (80.3%) were staff nurses and 34 (19.7%) were charge nurses or higher. Most participants had no prior experience of reporting child abuse (170, 98.3%) and had not received child abuse-related education (112, 64.7%) (Table 1). Notably, despite this heterogeneity in clinical experience and demographic background, none of these characteristics were significantly associated with reporting attitudes, as detailed below.

### 3.2. Differences in Study Variables According to General Characteristics

Table 2 presents the differences in child abuse knowledge, empathy, child abuse reporting self-efficacy, and attitudes toward reporting child abuse according to participants’ general characteristics.

Child abuse knowledge differed significantly according to the presence of children (t = 2.06, *p* = 0.041) and prior attendance of child abuse-related education (t = 3.08, *p* = 0.002). Participants with children (M = 40.53, SD = 4.13) had significantly higher knowledge scores than those without children (M = 39.09, SD = 5.01), and those who had attended child abuse education (M = 41.17, SD = 3.78) also had significantly higher knowledge scores than those who had not (M = 39.10, SD = 4.89). No significant differences in child abuse knowledge were found according to age (F = 2.44, *p* = 0.091), total clinical work experience (F = 2.13, *p* = 0.098), pediatric hospital work experience (F = 2.24, *p* = 0.067), marital status (F = 2.45, *p* = 0.089), position (t = −1.30, *p* = 0.197), or prior experience of reporting child abuse (t = −0.06, *p* = 0.956).

Empathy did not differ significantly according to any of the general characteristics examined (all *p* > 0.05). Similarly, child abuse reporting self-efficacy showed no statistically significant differences according to general characteristics (all *p* > 0.05).

Attitudes toward reporting child abuse did not differ significantly according to age (F = 3.02, *p* = 0.052), total clinical work experience (F = 1.78, *p* = 0.152), pediatric hospital work experience (F = 1.67, *p* = 0.167), marital status (F = 1.81, *p* = 0.168), presence of children (t = −0.33, *p* = 0.742), position (t = 0.73, *p* = 0.464), experience of reporting child abuse (t = 0.85, *p* = 0.396), or attendance of child abuse education (t = −0.02, *p* = 0.985).

### 3.3. Descriptive Statistics of Study Variables

Table 3 presents the descriptive statistics for the study variables and the subscale scores for each variable. The mean score for child abuse knowledge was 39.71 ± 4.74 out of a possible 49 points. The mean scores for child abuse reporting self-efficacy, empathy, and attitudes toward reporting child abuse were 3.35 ± 0.63 (out of 5), 2.79 ± 0.28 (out of 4), and 3.68 ± 0.49 (out of 5), respectively. Among the empathy subfactors, scores were highest for online simulation (2.94 ± 0.37) and lowest for emotion contagion (2.55 ± 0.53). For child abuse reporting self-efficacy, confidence in reporting suspected cases (3.51 ± 0.75) was higher than confidence in recognizing them (3.22 ± 0.64). Among the reporting-attitude subscales, advocacy for the child showed the most favorable scores (4.29 ± 0.55), whereas negative consequences of reporting scored lowest (3.35 ± 0.87).

### 3.4. Correlations Among Study Variables

Table 4 presents the correlations among the study variables. Attitudes toward reporting child abuse showed significant positive correlations with child abuse knowledge (r = 0.20, *p* = 0.009), child abuse reporting self-efficacy (r = 0.52, *p* < 0.001), and empathy (r = 0.28, *p* < 0.001). Child abuse knowledge was also positively correlated with child abuse reporting self-efficacy (r = 0.21, *p* = 0.006), and empathy was positively correlated with child abuse reporting self-efficacy (r = 0.27, *p* < 0.001). The correlation between child abuse knowledge and empathy was not statistically significant (r = 0.11, *p* = 0.138).

### 3.5. Indirect Associations Through Child Abuse Reporting Self-Efficacy

**Figure 1:**
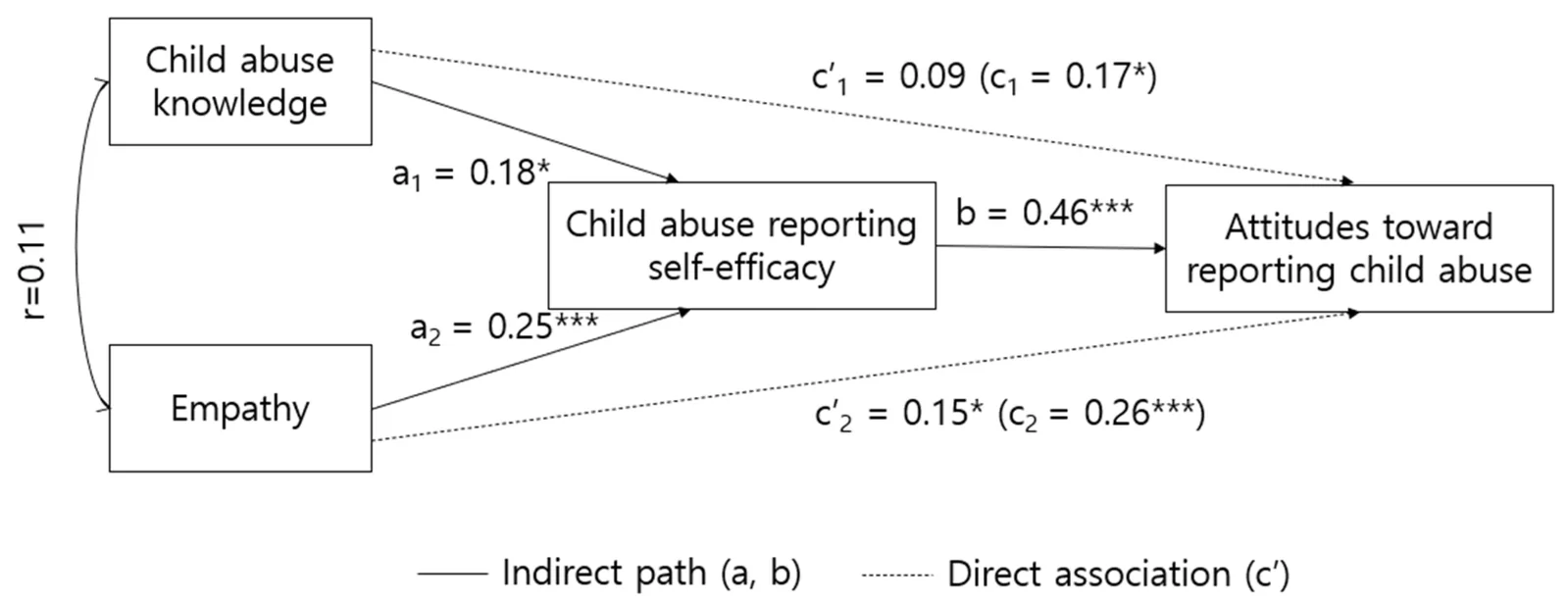
Integrated mediation model of the associations of child abuse knowledge and empathy with attitudes toward reporting child abuse through child abuse reporting self-efficacy (*n* = 173). Values are standardized coefficients (β). Solid lines represent the paths constituting the indirect associations (a, b), and dashed lines represent the direct effects (c′) of each predictor on attitudes after accounting for self-efficacy; total effects (c) are shown in parentheses. The curved double-headed arrow indicates the correlation between knowledge and empathy. Indirect association of knowledge: β = 0.08, 95% bootstrap CI [0.01, 0.15]; of empathy: β = 0.11, 95% bootstrap CI [0.03, 0.22]. Bootstrap CIs were based on 10,000 resamples. * *p* < 0.05, *** *p* < 0.001.

The results of the separate mediation models and the additional integrated mediation model are presented in Table 5, and the integrated model is illustrated in Figure 1. For each model, we report the total association (c) of the predictor with attitudes toward reporting child abuse, the direct association (c′) after accounting for child abuse reporting self-efficacy, and the indirect association through self-efficacy (the product of the a and b paths). Because of the cross-sectional study design, all mediation analyses were interpreted as tests of statistical indirect associations rather than causal mechanisms. Regression diagnostics indicated no evidence of multicollinearity (VIF = 1.05–1.12) or autocorrelation of residuals (Durbin–Watson = 1.89); all standardized residuals were within ±3, and no influential observations were identified (maximum Cook’s distance = 0.09).

Model 1: Child Abuse Knowledge → Child Abuse Reporting Self-Efficacy → Attitudes

In the separate mediation model, child abuse knowledge was significantly positively associated with child abuse reporting self-efficacy (a path: β = 0.21, t = 2.76, *p* = 0.006). Reporting self-efficacy was positively associated with attitudes toward reporting child abuse after accounting for knowledge (b path: β = 0.50, t = 7.42, *p* < 0.001). The total association between knowledge and reporting attitudes was significant (c: β = 0.20, t = 2.64, *p* = 0.009), whereas the direct association did not reach statistical significance after accounting for reporting self-efficacy (c′: B = 0.010, 95% CI [−0.004, 0.024]; β = 0.10, t = 1.43, *p* = 0.154).

The indirect association through reporting self-efficacy was statistically significant (unstandardized indirect effect B = 0.0107, BootSE = 0.0043, 95% bootstrap CI [0.0025, 0.0195]; completely standardized indirect effect β = 0.10, 95% bootstrap CI [0.02, 0.19]). However, the nonsignificant direct association should not be interpreted as evidence of full mediation or of the absence of a direct association, because its 95% CI included values comparable in size to the indirect association. These findings therefore indicate a significant indirect association between child abuse knowledge and reporting attitudes through reporting self-efficacy, without establishing a causal mediation mechanism or ruling out a direct association.

Model 2: Empathy → Child Abuse Reporting Self-Efficacy → Attitudes

In the separate mediation model, empathy was significantly positively associated with reporting self-efficacy (a path: β = 0.27, t = 3.65, *p* < 0.001). Reporting self-efficacy was positively associated with reporting attitudes after accounting for empathy (b path: β = 0.47, t = 7.04, *p* < 0.001). The total association between empathy and reporting attitudes was significant (c: β = 0.28, t = 3.85, *p* < 0.001), and the direct association remained statistically significant after accounting for reporting self-efficacy (c′: β = 0.16, t = 2.31, *p* = 0.022).

The indirect association through reporting self-efficacy was also statistically significant (unstandardized indirect effect B = 0.2207, BootSE = 0.0896, 95% bootstrap CI [0.0592, 0.4092]; completely standardized indirect effect β = 0.13, 95% bootstrap CI [0.03, 0.24]). Thus, empathy demonstrated both a significant direct association with reporting attitudes and a significant indirect association through reporting self-efficacy.

Additional Integrated Mediation and Sensitivity Analyses

To examine whether the indirect associations remained when child abuse knowledge and empathy were considered simultaneously, an integrated mediation model was estimated with both variables entered as predictors (Table 5 and Figure 1). Knowledge and empathy were weakly and nonsignificantly correlated (r = 0.11, *p* = 0.138). In the integrated model, both knowledge (a1: β = 0.18, t = 2.44, *p* = 0.016) and empathy (a2: β = 0.25, t = 3.40, *p* < 0.001) were independently associated with reporting self-efficacy, which in turn was positively associated with reporting attitudes (b: β = 0.46, t = 6.70, *p* < 0.001). The total associations with reporting attitudes were significant for both knowledge (c1: β = 0.17, *p* = 0.022) and empathy (c2: β = 0.26, *p* < 0.001). The direct association remained significant for empathy (c′2: β = 0.15, t = 2.23, *p* = 0.027) but did not reach statistical significance for knowledge (c′1: B = 0.009, 95% CI [−0.005, 0.023]; β = 0.09, t = 1.31, *p* = 0.192). The integrated model explained 29.5% of the variance in reporting attitudes (R^2^ = 0.295, F = 23.54, *p* < 0.001).

The indirect associations through child abuse reporting self-efficacy remained statistically significant for both knowledge (unstandardized indirect effect B = 0.0085, BootSE = 0.0037, 95% bootstrap CI [0.0013, 0.0161]; completely standardized indirect effect β = 0.08, 95% bootstrap CI [0.01, 0.15]) and empathy (B = 0.1970, BootSE = 0.0856, 95% bootstrap CI [0.0439, 0.3795]; β = 0.11, 95% bootstrap CI [0.03, 0.22]). Thus, the pattern observed in the separate models was maintained when both predictors were considered simultaneously.

The results of the sensitivity analyses, including the coefficients of the covariates, are presented in Table 6. Sensitivity analyses adjusting for total years of clinical experience and prior completion of child abuse-related education yielded consistent findings. After adjustment, the indirect associations through reporting self-efficacy remained statistically significant in the separate models for both knowledge (B = 0.0121, 95% bootstrap CI [0.0036, 0.0215]) and empathy (B = 0.2139, 95% bootstrap CI [0.0522, 0.4030]), as well as in the integrated model for both knowledge (B = 0.0098, 95% bootstrap CI [0.0020, 0.0181]) and empathy (B = 0.1847, 95% bootstrap CI [0.0335, 0.3677]). The pattern of significant and nonsignificant paths was unchanged; the direct association with reporting attitudes remained significant for empathy but not for knowledge. Neither clinical experience nor prior child abuse-related education was significantly associated with reporting self-efficacy or reporting attitudes in any of the adjusted models (all *p* > 0.10).

## 4. Discussion

This study aimed to identify the levels of child abuse knowledge, empathy, child abuse reporting self-efficacy, and attitudes toward reporting child abuse among nurses working in children’s hospitals, and to examine the indirect associations of knowledge and empathy with reporting attitudes through reporting self-efficacy. The mean score for child abuse knowledge was 39.71 out of a possible 49 points, somewhat higher than that reported in prior studies using the same instrument among nursing students [13]. Notably, this relatively high level of knowledge was not directly associated with reporting attitudes once self-efficacy was taken into account, a paradox addressed below. The mean empathy score was 2.79 out of a possible 4 points, lower than levels reported among nursing students and childcare teachers in prior studies [18,30]. Child abuse reporting self-efficacy was 3.35 out of a possible 5 points, similar to levels reported among emergency department nurses, childcare teachers, and elementary school teachers [4,27,28]. Attitudes toward reporting child abuse were 3.68 out of a possible 5 points, an intermediate level compared with values reported among emergency department nurses and clinical nurses in prior studies [12,13]. These findings suggest that, although nurses working in children’s hospitals reported a relatively high level of knowledge and a favorable attitude toward reporting child abuse, only three of the 173 nurses had ever reported suspected abuse, and their empathy scores were lower than those reported in some other samples. Furthermore, the clinical circumstances in which nurses encounter suspected child abuse may differ between emergency departments and children’s hospitals. Nurses in children’s hospitals may encounter ambiguous circumstances and indirect signs of suspected abuse during routine clinical care, potentially presenting challenges in recognizing and responding to such cases [28]. Prior studies suggest that workload, time pressure, and emotional labor may be associated with lower empathy among nurses [29]. However, these factors were not measured in the present study, and their potential contribution to differences in empathy should be regarded as a hypothesis based on previous literature rather than a finding of this study. Further research examining organizational and emotional factors is needed to clarify these relationships.

Attitudes toward reporting child abuse did not differ significantly according to general characteristics, including age, total clinical work experience, work experience in a children’s hospital, marital status, presence of children, position, prior experience reporting child abuse, and attendance of child abuse-related education. This finding differs from that of prior studies [30]. One possible explanation is that reporting attitudes in this sample may be shaped less by individual characteristics than by factors that were not measured here, such as organizational environment or psychological factors; this remains a hypothesis for future research.

Child abuse knowledge, empathy, and child abuse reporting self-efficacy were positively correlated with attitudes toward reporting child abuse. This is consistent with prior studies that have regarded knowledge as an antecedent of reporting-related behavioral change [12,14]. The association between child abuse knowledge and reporting attitudes is compatible with the view that a greater understanding of the definition, types, indicators, reporting procedures, and legal aspects of child abuse may heighten sensitivity to abusive situations and the willingness to actively intervene. The finding that child abuse reporting self-efficacy was significantly associated with both child abuse knowledge and empathy suggests that confidence in one’s ability to report may be higher among nurses with greater knowledge or empathy. Because these data are cross-sectional, such associations do not establish the direction of effects. As attempts to explain child abuse reporting behavior increasingly incorporate psychological and emotional variables such as empathy and moral sensitivity, the present findings support the need to consider these psychological and emotional factors together with cognitive factors.

The analysis of indirect associations through child abuse reporting self-efficacy showed that child abuse knowledge had a statistically significant indirect association with attitudes toward reporting child abuse through reporting self-efficacy, whereas its direct association was not statistically significant after accounting for self-efficacy. However, the nonsignificant direct association does not establish the absence of a direct relationship; its confidence interval included values comparable in size to the indirect association, and the study may have had limited power to detect a small direct association. In contrast, empathy showed both significant direct and indirect associations with reporting attitudes. These patterns were maintained in the integrated model, in which knowledge and empathy were entered simultaneously, and after adjustment for clinical experience and prior child abuse-related education, indicating that the indirect association of each predictor was not attributable to its overlap with the other or to these covariates. These findings suggest that reporting self-efficacy may be an important correlate of reporting attitudes in both models, although the cross-sectional design does not establish causal mediation. That is, greater child abuse knowledge and empathy were associated with greater child abuse reporting self-efficacy among nurses, which, in turn, was associated with more favorable attitudes toward reporting child abuse. In the knowledge model, the indirect association through reporting self-efficacy was statistically significant, while the direct association was not. In the empathy model, both direct and indirect associations were statistically significant. This is consistent with prior domestic and international studies indicating that child abuse reporting self-efficacy is an important correlate of both reporting intention and reporting attitude, which have been examined as precursors of reporting behavior [4,27,28]. However, reporting intentions, reporting attitudes, and actual reporting behavior are distinct outcomes. Child abuse reporting self-efficacy—the belief that one can judge a suspected case of abuse and report it through the appropriate procedures—may be relevant to overcoming perceived barriers to reporting, as suggested by theory and previous literature. Nevertheless, the present study did not examine whether reporting self-efficacy translates into actual reporting behavior. Empathy may also be associated with favorable reporting attitudes by supporting sensitivity to children’s suffering and vulnerability and a sense of professional responsibility for child protection [19,31].

Accordingly, favorable attitudes toward reporting child abuse may be associated not only with knowledge of legal obligations but also with reporting self-efficacy and empathy. Our findings are consistent with the conceptual distinction—central to Bandura’s self-efficacy theory—between knowing and being able to act [17]. Child abuse knowledge may provide a cognitive foundation for recognizing suspected abuse, while reporting self-efficacy reflects confidence in carrying out the reporting process. However, the present findings do not establish that self-efficacy is a necessary step linking knowledge to reporting attitudes. By contrast, empathy retained a significant direct association with attitudes toward reporting child abuse even after controlling for child abuse reporting self-efficacy, indicating that its association with reporting attitudes was not fully accounted for by self-efficacy in the model examined. Emotional engagement and moral motivation may help explain this association, although these potential mechanisms were not directly tested. Thus, the findings suggest that child abuse knowledge and empathy may exhibit different patterns of association with reporting attitudes. Reporting self-efficacy was involved in significant indirect associations in both models, while a statistically significant direct association with reporting attitudes was observed only for empathy. These findings should be interpreted as statistical associations rather than evidence of distinct causal mechanisms.

The low proportion of child abuse reports submitted by healthcare professionals in South Korea underscores the importance of further research on factors associated with reporting practices. Although the present findings identify reporting self-efficacy as a correlate of reporting attitudes, they do not establish the reasons for low reporting rates or explain actual reporting behavior. Notably, public and media attention to child abuse in Korea increased following high-profile cases, including the 2020 death of an adopted infant; consistent with this shift in public attention, Lee et al. [32] reported that approximately 60% of child abuse-related news articles in their sample of major Korean newspapers published between 2018 and 2021 appeared in 2020–2021 alone. However, changes in media attention cannot be directly linked to nurses’ reporting attitudes or reporting practices based on the present data. Future research should examine whether reporting confidence, organizational factors, and other potential barriers are associated with actual reporting behavior. International child abuse reporting statistics should be interpreted with caution because child protection systems, mandatory reporting requirements, reporting thresholds, and recording practices differ across countries. The Korean figure (<1%) reflects the share of all child abuse reports made by healthcare professionals [3], whereas the U.S. figure (about 26%) reflects the share of infant child-welfare investigations originating from medical professionals’ reports [6], and the Australian figure (14%) reflects the share of known notification sources attributed to medical or health personnel [7]. Because these indicators differ in their populations and denominators, they cannot be used to draw direct conclusions about differences in reporting performance across countries. Nevertheless, these statistics highlight the involvement of healthcare professionals in child protection systems. The present findings suggest that future educational approaches could consider reporting confidence alongside child abuse knowledge, although the effectiveness of such approaches remains to be established. Therefore, future child abuse-related education programs and institutional interventions could go beyond knowledge transmission and test an integrated strategy that targets child abuse reporting self-efficacy through simulation- and case-based learning reflecting real clinical situations, explores the potential role of organizational support and emotional exhaustion, and includes programs to enhance empathy. Whether such approaches improve attitudes toward reporting or actual reporting behavior must be verified in longitudinal or intervention studies.

### 4.1. Strengths

This study has several notable strengths. First, to our knowledge, this is among the first studies to simultaneously examine the indirect associations of two distinct predictors—cognitive (child abuse knowledge) and affective (empathy)—with attitudes toward reporting child abuse through child abuse reporting self-efficacy among nurses working in children’s hospitals, a population uniquely positioned as frontline mandated reporters. Second, child abuse knowledge and empathy were examined both in separate models, reflecting their theoretically distinct roles, and in an integrated model in which both predictors were entered simultaneously. The consistency of the findings across these specifications suggests that the indirect association of each predictor was not attributable to its overlap with the other. Third, the statistical significance of the indirect effects was evaluated using percentile bootstrapping with 10,000 resamples, which does not rely on the normality assumption required by the traditional Sobel test and is considered a more robust and recommended approach for testing indirect associations in behavioral and health science research. Fourth, the robustness of the mediation findings was further supported by sensitivity analyses controlling for years of clinical experience and prior completion of child abuse-related education in both the separate and integrated models; the indirect associations remained statistically significant, and the pattern of a significant direct association for empathy and a nonsignificant direct association for knowledge was unchanged after adjustment, suggesting that the findings were not explained by these measured covariates. Fifth, by examining differences in reporting attitudes across a comprehensive set of participants’ general characteristics, this study found that reporting attitudes did not vary significantly by age, clinical experience, marital status, or position, suggesting that child abuse reporting self-efficacy—a potentially modifiable factor—may be a promising focus for future educational and clinical interventions, although its effectiveness as an intervention target remains to be tested. Finally, all study measures demonstrated acceptable to excellent internal consistency (KR-20 = 0.77 for knowledge; Cronbach’s α = 0.83–0.90 for the other measures), supporting the reliability of the study findings.

### 4.2. Limitations and Recommendations for Further Research

This study has several limitations. First, the cross-sectional design precludes any causal interpretation of the observed relationships. Although the hypothesized directional pathways were grounded in theory, the temporal precedence of child abuse knowledge, empathy, child abuse reporting self-efficacy, and attitudes toward reporting child abuse could not be established, and reverse or reciprocal relationships could not be ruled out. Accordingly, the mediation results should be interpreted as statistical indirect associations rather than causal mechanisms. Second, all study variables were measured using self-report questionnaires administered at a single time point, which may have introduced common method and social desirability biases, particularly for sensitive topics such as child abuse reporting. Third, the sample was drawn from nurses working in seven children’s hospitals in a single metropolitan area using convenience sampling, which limits the generalizability of the findings to nurses working in other geographic regions or clinical settings. In addition, because hospital affiliation was not recorded to protect participant anonymity, the potential clustering of participants within the seven hospitals could not be accounted for, and nonindependence of observations within hospitals may have led to underestimated standard errors. Furthermore, only three participants (1.7%) reported previous experience with child abuse reporting. Importantly, this study measured self-reported attitudes toward reporting child abuse rather than actual reporting behavior. Therefore, the findings should be interpreted as evidence of associations with reporting attitudes and should not be considered evidence of changes or improvements in actual reporting practices. Notably, none of the participants’ general characteristics were significantly associated with attitudes toward reporting child abuse, although this finding does not necessarily indicate that the sample was homogeneous in reporting attitudes. Fourth, although the sample size (*n* = 173) was adequate for the bootstrap mediation analyses, it may have had limited statistical power to detect smaller effects, particularly the direct association of child abuse knowledge with reporting attitudes. Thus, the nonsignificant direct association of knowledge with reporting attitudes should not be interpreted as indicating the absence of a direct association, as its confidence interval was compatible with a small direct association. Fifth, child abuse knowledge and empathy were initially examined as predictors of reporting attitudes in two separate mediation models to examine the distinct theoretical roles of knowledge as a cognitive factor and empathy as an affective factor. Although an additional integrated model in which both predictors were entered simultaneously yielded findings consistent with those of the separate models, the weak and nonsignificant correlation between the two predictors (r = 0.11) does not establish their independence, and neither the separate nor the integrated models can exclude confounding by unmeasured variables that may be related to both knowledge and empathy. In addition, because knowledge and empathy were measured on different scales, their relative contributions to reporting attitudes should be compared with caution. Finally, although sensitivity analyses controlling for years of clinical experience and prior completion of child abuse-related education in both the separate and integrated models yielded highly consistent results, other potentially relevant factors could not be examined. In particular, prior experience of reporting child abuse could not be included as a covariate because of the extreme response imbalance in this sample (only three of 173 participants reported prior reporting experience), and unmeasured factors such as institutional reporting protocols or prior direct exposure to suspected child abuse cases may also influence the relationships examined and warrant further investigation with larger and more diverse samples. Future longitudinal and intervention studies involving geographically diverse samples are needed to examine whether interventions to strengthen child abuse reporting self-efficacy are associated with improvements in reporting attitudes and lead to changes in actual reporting behavior. Such studies should also examine organizational support and emotional labor as potential moderators of the relationship between empathy and attitudes toward reporting child abuse.

## 5. Conclusions

This study examined the relationships among child abuse knowledge, empathy, child abuse reporting self-efficacy, and attitudes toward reporting child abuse among 173 nurses working in children’s hospitals, and identified significant indirect associations of child abuse knowledge and empathy with reporting attitudes through child abuse reporting self-efficacy. In the knowledge model, the indirect association through reporting self-efficacy was statistically significant, whereas the direct association was not statistically significant after accounting for self-efficacy. In the empathy model, both direct and indirect associations with reporting attitudes were statistically significant. These patterns were consistent in an integrated model including both predictors and after adjustment for clinical experience and prior child abuse-related education.

These findings suggest that child abuse reporting self-efficacy may be an important correlate of favorable attitudes toward reporting child abuse, alongside knowledge and empathy. However, because this study measured self-reported reporting attitudes rather than actual reporting behavior, the findings should not be interpreted as evidence of improvements in actual reporting practices.

Simulation-based education, practical training in child abuse reporting procedures, and institutional support may be considered potential strategies for strengthening reporting self-efficacy and promoting favorable reporting attitudes. Nevertheless, the effectiveness of these approaches has not been established by the present study. Future longitudinal and intervention studies are needed to determine whether such strategies improve reporting attitudes and lead to changes in actual reporting behavior.

## Figures and Tables

**Table 1:** Characteristics of participants (*n* = 173).

Characteristic	Categories	*n*	%	Mean ± SD
Age (year)	≤29	37	21.4	36.92 ± 8.03
	30~39	62	35.8	
	≥40	74	42.8	
Total Clinical Work Experience (year)	<5	39	22.5	11.45 ± 7.46
	5~10	38	22.0	
	10~<15	36	20.8	
	≥15	60	34.7	
Pediatric Hospital Work Experience (year)	<3	49	28.3	8.29 ± 6.89
	3~<5	24	13.9	
	5~<10	33	19.1	
	10~<15	26	15.0	
	≥15	41	23.7	
Marital Status	Single	69	39.9	
	Married	99	57.2	
	Divorced	5	2.9	
Presence of Children	Yes	88	50.9	
	No	85	49.1	
Position	Staff Nurse	139	80.3	
	Charge Nurse or Higher	34	19.7	
Experience of Reporting Child Abuse	Yes	3	1.7	
	No	170	98.3	
Attendance of Child Abuse Education	Yes	61	35.3	
	No	112	64.7	

**Table 2:** Differences in child abuse knowledge, empathy, self-efficacy, and reporting attitudes by participants’ characteristics (*n* = 173).

Characteristic	Categories	Child Abuse Knowledge		Empathy		Child Abuse Reporting Self-Efficacy		Attitudes Toward Child Abuse Reporting	
		M ± SD	t/F(*p*) Scheffé	M ± SD	t/F(*p*) Scheffé	M ± SD	t/F(*p*) Scheffé	M ± SD	t/F(*p*) Scheffé
Age (year)	≤29	39.03 ± 4.43	2.44 (0.091)	2.83 ± 0.33	0.59 (0.556)	3.41 ± 0.73	0.23 (0.793)	3.86 ± 0.51	3.02 (0.052)
	30~39	39.23 ± 4.87		2.79 ± 0.27		3.38 ± 0.54		3.63 ± 0.51	
	≥40	40.70 ± 4.43		2.77 ± 0.27		3.33 ± 0.61		3.64 ± 0.46	
Total Clinical Work Experience (year)	<5	39.13 ± 5.06	2.13 (0.098)	2.85 ± 0.35	0.74 (0.532)	3.48 ± 0.65	1.76 (0.157)	3.84 ± 0.52	1.78 (0.152)
	5~<10	39.47 ± 4.14		2.77 ± 0.26		3.34 ± 0.63		3.67 ± 0.54	
	10~<15	38.94 ± 5.27		2.78 ± 0.27		3.48 ± 0.60		3.60 ± 0.51	
	≥15	41.00 ± 4.07		2.77 ± 0.26		3.24 ± 0.57		3.63 ± 0.43	
Work Experience in a Children’s hospital (year)	<3	38.35 ± 5.06	2.24 (0.067)	2.81 ± 0.30	0.60 (0.667)	3.49 ± 0.60	1.72 (0.148)	3.80 ± 0.56	1.67 (0.167)
	3~<5	40.48 ± 4.26		2.86 ± 0.28		3.50 ± 0.65		3.75 ± 0.37	
	5~<10	39.50 ± 4.90		2.75 ± 0.28		3.21 ± 0.69		3.61 ± 0.51	
	10~<15	40.77 ± 4.27		2.77 ± 0.26		3.39 ± 0.63		3.66 ± 0.55	
	≥15	40.85 ± 3.95		2.77 ± 0.28		3.26 ± 0.50		3.57 ± 0.40	
Marital Status	Single	38.91 ± 5.13	2.45 (0.089)	2.76 ± 0.30	0.58 (0.561)	3.37 ± 0.63	0.12 (0.988)	3.75 ± 0.54	1.81 (0.168)
	Married	40.49 ± 4.17		2.81 ± 0.27		3.37 ± 0.61		3.64 ± 0.46	
	Divorced	39.20 ± 4.71		2.76 ± 0.18		3.33 ± 0.56		3.40 ± 0.32	
Presence of Children	Yes	40.53 ± 4.13	2.06 (0.041)	2.81 ± 0.26	0.70 (0.487)	3.40 ± 0.64	0.75 (0.457)	3.67 ± 0.48	−0.33 (0.742)
	No	39.09 ± 5.01		2.78 ± 0.30		3.33 ± 0.59		3.69 ± 0.52	
Position	Staff nurse	39.59 ± 4.70	−1.30 (0.197)	2.80 ± 0.28	0.58 (0.560)	3.39 ± 0.64	1.08 (0.282)	3.69 ± 0.51	0.73 (0.464)
	Charge nurse or higher	40.74 ± 4.30		2.76 ± 0.28		3.26 ± 0.48		3.62 ± 0.42	
Experience of Reporting Child abuse	Yes	39.67 ± 6.66	−0.06 (0.956)	2.78 ± 0.14	−0.05 (0.960)	3.58 ± 0.69	0.62 (0.538)	3.92 ± 0.85	0.85 (0.396)
	No	39.82 ± 4.62		2.79 ± 0.28		3.36 ± 0.61		3.68 ± 0.49	
Attendance of Child Abuse Education	Yes	41.17 ± 3.78	3.08 (0.002)	2.79 ± 0.26	0.07 (0.941)	3.33 ± 0.61	−0.55 (0.585)	3.68 ± 0.50	−0.02 (0.985)
	No	39.10 ± 4.89		2.79 ± 0.29		3.39 ± 0.62		3.68 ± 0.49	

**Table 3:** Descriptive statistics of research variables (*n* = 173).

Variable	*n*	Mean ± SD	Item Range	Range
Child abuse knowledge	49	39.71 ± 4.74	23.00~47.00	0~49
Knowledge of child abuse and reporting	15	10.61 ± 1.78	3.00~14.00	0~15
Knowledge of evidence of child abuse	34	29.10 ± 3.97	15.00~34.00	0~34
Empathy	26	2.79 ± 0.28	2.04~3.85	1~4
Perspective taking	10	2.82 ± 0.34	1.60~4.00	
Online simulation	6	2.94 ± 0.37	1.00~4.00	
Emotion contagion	4	2.55 ± 0.53	1.00~4.00	
Peripheral responsivity	3	2.70 ± 0.57	1.00~4.00	
Proximal responsivity	3	2.80 ± 0.44	1.33~4.00	
Child abuse reporting self-efficacy	8	3.35 ± 0.63	1.00~5.00	1~5
Confidence in recognition	4	3.22 ± 0.64	1.00~5.00	
Confidence in reporting	4	3.51 ± 0.75	1.00~5.00	
Attitudes toward reporting child abuse	17	3.68 ± 0.49	2.65~4.82	1~5
Negative consequences of reporting	4	3.35 ± 0.87	1.00~5.00	
Impact on child after reporting	4	3.61 ± 0.67	1.75~5.00	
Discomfort with reporting	4	3.36 ± 0.79	1.25~5.00	
Advocacy for the child	2	4.29 ± 0.55	3.00~5.00	
Avoidance of responsibility in reporting	3	4.23 ± 0.70	1.00~5.00	

**Table 4:** Correlations among study variables (*n* = 173).

Variables	Child Abuse Knowledge	Empathy	Child Abuse Reporting Self-Efficacy	Attitudes Toward Reporting Child Abuse
	r(*p*)	r(*p*)	r(*p*)	r(*p*)
Child Abuse Knowledge	1			
Empathy	0.11(0.138)	1		
Child Abuse Reporting Self-efficacy	0.21(0.006)	0.27(<0.001)	1	
Attitudes Toward Reporting Child Abuse	0.20(0.009)	0.28(<0.001)	0.52(<0.001)	1

**Table 5:** Separate and integrated mediation models of child abuse reporting self-efficacy in the associations of child abuse knowledge and empathy with attitudes toward reporting child abuse (*n* = 173).

Model/Path	B	SE	β	t	*p*	95% CI of B (LL, UL)
Model 1: Knowledge (separate model)						
Knowledge → Self-efficacy (a)	0.028	0.010	0.21	2.76	0.006	0.008, 0.047
Self-efficacy → Attitudes (b)	0.388	0.052	0.50	7.42	<0.001	0.284, 0.491
Knowledge → Attitudes, total (c)	0.021	0.008	0.20	2.64	0.009	0.005, 0.036
Knowledge → Attitudes, direct (c′)	0.010	0.007	0.10	1.43	0.154	−0.004, 0.024
Indirect: Knowledge → Self-efficacy → Attitudes	0.0107	0.0043	0.10 [0.02, 0.19]			0.0025, 0.0195
Self-efficacy: R^2^ = 0.043, F = 7.62, *p* = 0.006; Attitudes: R^2^ = 0.274, F = 32.08, *p* < 0.001						
Model 2: Empathy (separate model)						
Empathy → Self-efficacy (a)	0.596	0.163	0.27	3.65	<0.001	0.273, 0.918
Self-efficacy → Attitudes (b)	0.370	0.053	0.47	7.04	<0.001	0.267, 0.474
Empathy → Attitudes, total (c)	0.490	0.127	0.28	3.85	<0.001	0.238, 0.741
Empathy → Attitudes, direct (c′)	0.269	0.117	0.16	2.31	0.022	0.039, 0.499
Indirect: Empathy → Self-efficacy → Attitudes	0.2207	0.0896	0.13 [0.03, 0.24]			0.0592, 0.4092
Self-efficacy: R^2^ = 0.072, F = 13.30, *p* < 0.001; Attitudes: R^2^ = 0.288, F = 34.31, *p* < 0.001						
Integrated model: Knowledge and empathy entered simultaneously						
Knowledge → Self-efficacy (a)	0.024	0.010	0.18	2.44	0.016	0.005, 0.043
Empathy → Self-efficacy (a)	0.551	0.162	0.25	3.40	<0.001	0.231, 0.871
Self-efficacy → Attitudes (b)	0.358	0.053	0.46	6.70	<0.001	0.252, 0.463
Knowledge → Attitudes, total (c)	0.018	0.008	0.17	2.30	0.022	0.003, 0.033
Knowledge → Attitudes, direct (c′)	0.009	0.007	0.09	1.31	0.192	−0.005, 0.023
Empathy → Attitudes, total (c)	0.457	0.127	0.26	3.61	<0.001	0.207, 0.707
Empathy → Attitudes, direct (c′)	0.260	0.117	0.15	2.23	0.027	0.029, 0.490
Indirect: Knowledge → Self-efficacy → Attitudes	0.0085	0.0037	0.08 [0.01, 0.15]			0.0013, 0.0161
Indirect: Empathy → Self-efficacy → Attitudes	0.1970	0.0856	0.11 [0.03, 0.22]			0.0439, 0.3795
Self-efficacy: R^2^ = 0.104, F = 9.82, *p* < 0.001; Attitudes: R^2^ = 0.295, F = 23.54, *p* < 0.001						

SE, standard error; CI, confidence interval. Values are standardized regression coefficients (B) for direct effects, and unstandardized effect sizes for indirect and total effects, consistent with Hayes’ PROCESS macro output.

**Table 6:** Indirect associations through child abuse reporting self-efficacy before and after adjustment for potential confounders (*n* = 173).

Model	Predictor	Unadjusted: a (β)	b (β)	c′ (β)	Indirect B [95% CI]	Adjusted: a (β)	b (β)	c′ (β)	Indirect B [95% CI]
Model 1 (separate)	Knowledge	0.21 **	0.50 ***	0.10	0.0107 [0.0025, 0.0195]	0.24 **	0.48 ***	0.11	0.0121 [0.0036, 0.0215]
Model 2 (separate)	Empathy	0.27 ***	0.47 ***	0.16 *	0.2207 [0.0592, 0.4092]	0.26 ***	0.47 ***	0.15 *	0.2139 [0.0522, 0.4030]
Integrated model	Knowledge	0.18 *	0.46 ***	0.09	0.0085 [0.0013, 0.0161]	0.21 **	0.45 ***	0.10	0.0098 [0.0020, 0.0181]
	Empathy	0.25 ***	0.46 ***	0.15 *	0.1970 [0.0439, 0.3795]	0.24 **	0.45 ***	0.14 *	0.1847 [0.0335, 0.3677]
**Covariates in the adjusted models, β** (***p***)									
Clinical experience → Self-efficacy			Model 1: −0.13 (0.107)		Model 2: −0.08 (0.300)		Integrated: −0.10 (0.170)		
Clinical experience → Attitudes			Model 1: −0.09 (0.189)		Model 2: −0.07 (0.309)		Integrated: −0.08 (0.229)		
Child abuse education → Self-efficacy			Model 1: −0.06 (0.443)		Model 2: −0.02 (0.819)		Integrated: −0.06 (0.446)		
Child abuse education → Attitudes			Model 1: 0.02 (0.764)		Model 2: 0.04 (0.560)		Integrated: 0.02 (0.772)		

a = predictor → child abuse reporting self-efficacy; b = self-efficacy → attitudes toward reporting child abuse; c′ = direct association of the predictor with attitudes; β = standardized coefficient; B = unstandardized indirect effect; CI = percentile bootstrap confidence interval (10,000 resamples). Adjusted models included total years of clinical experience (continuous) and prior completion of child abuse-related education (1 = yes, 0 = no) as covariates in both the mediator equation (self-efficacy) and the outcome equation (attitudes). In the integrated model, knowledge and empathy were entered simultaneously in both equations. * *p* < 0.05, ** *p* < 0.01, *** *p* < 0.001.

## Data Availability

The data presented in this study are available on request from the corresponding author due to institutional data protection policies protecting participant confidentiality.
